# Le syndrome de Senior Loken

**DOI:** 10.11604/pamj.2015.22.141.8042

**Published:** 2015-10-14

**Authors:** Manel Jellouli, Tahar Gargah

**Affiliations:** 1 Service de Pédiatrie, Hôpital Charles Nicolle, Tunis, Tunisie

**Keywords:** Senior Loken, nephronophtise, insuffisance rénale, Senior Loken, nephronophthisis, renal failure

## Image en medicine

**Figure 1:**
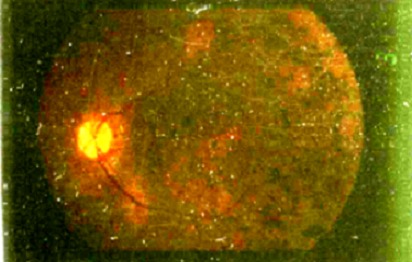
Fond d’œil montrant une pâleur papillaire, un aspect en sel et poivre caractéristique du syndrome de Sénior Loken

Le syndrome de Sénior Loken est une ciliopathie très rare à transmission autosomique récessive caractérisée par l'association d'une néphropathie tubulo-interstitielle chronique, la néphronophtise, avec une dystrophie rétinienne. M âgé de 7 ans, issu d'un mariage consanguin du premier degré était hospitalisé pour prise en charge d'une insuffisance rénale chronique. Sa sœur est en insuffisance rénale terminale sous hémodialyse et présente par ailleurs une cécité. Le patient présentait depuis quelque année une polyurie. L'examen objectivait un retard de croissance avec un poids de 18 kg et une taille de 105 cm (<-2 DS), la tension artérielle était à 110/70 mmHg, les urines étaient hypotoniques avec absence d'hématurie et de protéinurie à l'examen à la bandelette urinaire. A la biologie, l'hémoglobine était à 5 g/dl, la créatinine sanguine était à 350 µmol/L, la natrémie à 129 mmol/L et la kaliémie à 3,3 mmol/L. L’échographie rénale montrait des reins de petite taille à cortex hyperéchogène avec des kystes médullaires. Il présentait par ailleurs une baisse progressive de la vision. L'examen ophtalmologique a objectivé un nystagmus bilatéral avec un segment antérieur normal, au fond d’œil on notait une pâleur papillaire, des vaisseaux grèles et une rétinite pigmentaire avec l'aspect en sel et poivre (figure 1). L’électrorétinogramme a détecté une dégénerescence tapéto-rétinienne confirmant le diagnostic de Sénior Loken. L'audiogramme a objectivé une surdité de perception bilatérale. L’évolution était marquée par la perte de la vue à l’âge de 12 ans et le passage en insuffisance rénale terminale traité par hémodialyse.

